# Single Cell Dynamics Causes Pareto-Like Effect in Stimulated T Cell Populations

**DOI:** 10.1038/srep17756

**Published:** 2015-12-09

**Authors:** Jérémie Cosette, Alice Moussy, Fanny Onodi, Adrien Auffret-Cariou, Thi My Anh Neildez-Nguyen, Andras Paldi, Daniel Stockholm

**Affiliations:** 1 Genethon, 1bis rue de l’Internationale, 91000 Evry, France; 2 Unit INSERM INTEGRARE UMRS 951-UEVE-EPHE, 91000 Evry, France; 3 Ecole Pratique des Hautes Etudes, Paris, France

## Abstract

Cell fate choice during the process of differentiation may obey to deterministic or stochastic rules. In order to discriminate between these two strategies we used time-lapse microscopy of individual murine CD4 + T cells that allows investigating the dynamics of proliferation and fate commitment. We observed highly heterogeneous division and death rates between individual clones resulting in a Pareto-like dominance of a few clones at the end of the experiment. Commitment to the Treg fate was monitored using the expression of a GFP reporter gene under the control of the endogenous Foxp3 promoter. All possible combinations of proliferation and differentiation were observed and resulted in exclusively GFP–, GFP+ or mixed phenotype clones of very different population sizes. We simulated the process of proliferation and differentiation using a simple mathematical model of stochastic decision-making based on the experimentally observed parameters. The simulations show that a stochastic scenario is fully compatible with the observed Pareto-like imbalance in the final population.

Naïve CD4 + T lymphocytes are able to take multiple fate-decisions; they can give rise to various specialized cell types such as T helper effector or regulatory T (Treg) lymphocytes^1^. They do this in response to stimulations of their T-cell receptors (TCR) and various cytokines. Although studied for decades, the mechanisms of cell fate choice between different options remain elusive. The hypothesis of stochastic fate choice of hematopoietic cells was proposed 50 (fifty!) years ago^2^. Yet, the debate between the stochastic and deterministic mechanisms is still not settled. Some consider that the acquisition of the differentiated phenotype occurs via a predetermined pathway^3^, where each signal induces a defined cell fate. Others argue in favour of a stochastic mechanism^4^. According to this view, a cell responds to a signal by randomly choosing between two or more options. It is the collective action of the individual stochastic choices that creates non-random regularities at the level of the whole cell population. We have previously observed that phenotypic heterogeneity may appear spontaneously and contribute to the fate decisions in a clonal population without the action of external signals^5^,^6^,^7^,^8^. In the present study we aimed to evaluate the stochastic contribution to T cell differentiation on the basis of single-cell observations obtained in an *in vitro* system. When the naïve T cells are stimulated *in vitro* with anti-CD3 and anti-CD28 antibody-coated beads, IL-2 and TGF-β, they proliferate and preferentially acquire the Treg phenotype making this artificial system convenient for the study of cell fate decision-making mechanisms^9^. These conditions are highly selective, because essentially all cells acquire Treg phenotype after a week or so of culture. One can therefore consider that there is little room left for chance during this process. Nevertheless, the cells take at least two different decisions: they divide or they differentiate. It is not known whether these two decisions are independent or whether they are taken in a fixed pre-determined order. Recently, we observed that the majority of the cells are displaying a Treg phenotype after a week of culture, some cells reach this stage after only one or two divisions while others divide up to ten times^7^. This substantial proliferation heterogeneity is surprising in a cell population where each cell encounters identical conditions.

In order to get insight in the origin of this heterogeneous behavior we used a single-cell time-lapse approach coupled to mathematical modeling. Single-cell observations were successfully used to demonstrate the stochastic nature of fate decisions in B-cell differentiation^10^. Here, we used primary CD4 + cells from Foxp3-GFP knock-in mice so the acquisition of the Treg phenotype could be monitored in living cells using the expression of the GFP protein^11^. We observed substantial heterogeneity in the proliferation, differentiation and death rates leading to an unequal contribution of clonal cell lineages to the final population. Data-driven modeling of stochastic cell decision allowed us to show that the observed Pareto-like effect essentially results from the cumulative effect of stochastic cell decisions and events. Variations of cell cycle length and cell death rate are the key factors contributing to the phenotypic heterogeneity of the final cell population. The initial differences between the cells in the starting population may reinforce this effect but alone is insufficient to fully account for it.

Our observations show that due to the heterogeneity of proliferation and death rate, the final cell population is composed essentially from cells derived from a small number of initial founder cells.

## Results

### Time-lapse microscopy enables the exploration of single cell fate and shows that different scenarios of clonal differentiation/proliferation co-exist

**Figure 1:**
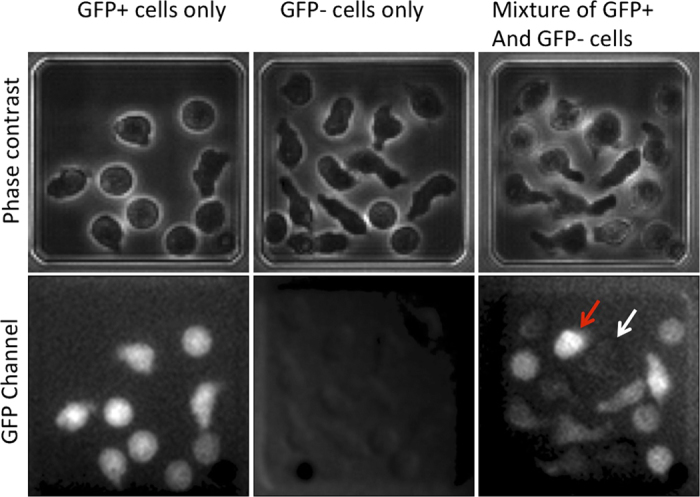
Cell division and phenotype detection using phase-contrast and fluorescent imaging. Phase contrast and green fluorescence channel representative images of the three main scenarios are observed: homogenous GFP+ Treg phenotype, homogenous GFP– phenotype and heterogeneous mixture of GFP+ (red arrow) and GFP– (white arrow) phenotypes.

Individual micro-wells containing a single cell were imaged every hour over a period of a hundred hours using time-lapse microscopy. We used the phase contrast channel to identify the cells, detect and count divisions, and detect the presence of the activation bead (Fig. 1). The fluorescence channel (GFP) was used to monitor the GFP fluorescence level that indicated the expression of the Foxp3 gene. The cells expressing GFP were considered as a sign of fate decision to acquire the Treg phenotype. No functional analyses were performed on these cells in this study. However, identical conditions were used in our previous work where the functional characteristics of these cells were confirmed^7^. Cell death was detected with the help of different parameters: the shape on the phase contrast image, and strong auto-fluorescence in the Rhodamine channel (not shown).

**Figure 2:**
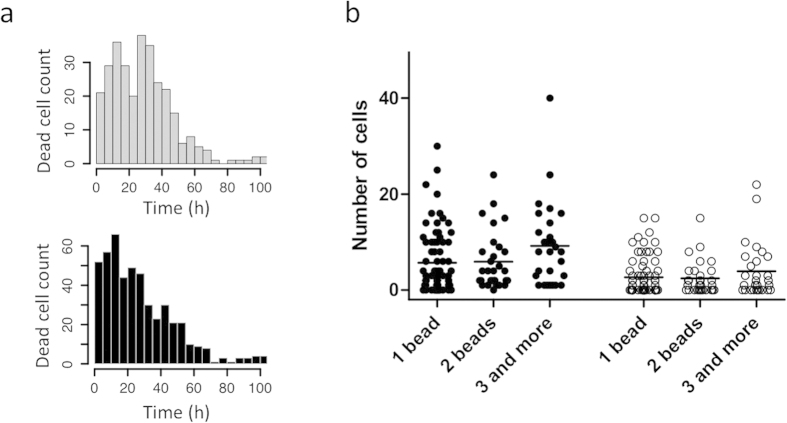
Influence of cell stimulation on mortality and proliferation. (**a**) Distribution of cell mortality during the experiment in absence (upper panel) and in presence (lower panel) of an activating bead in the micro-well shows no statistical difference (Kolmogorov-Smirnoff test p = 0.9952). (**b**) Clone size distribution (left – black dots) and number of GFP+ cells per clone (right – white dots) at the end of the experiment (100 h) in the presence of 1, 2 or 3 + beads. Each dot represents a single clone. The non-parametric ANOVA Kruskal-Wallis test was used to compare the different groups. Neither the total cell number (p = 0.0847) nor the number of GFP+ cells (p = 0.5529) were found significantly different. Same conclusion was reached using the Dunn’s multiple comparison test.

High rate of mortality was observed during the first 24 hours. In the 630 micro-wells containing a single living cell and at least one activation bead at t = 0h, 260 (41%) cells died during this period (Fig. 2). In the 310 micro-wells containing a single cell but no beads 131 cells (42%) died during the first 24 hours of culture. There was no significant difference in the death rate between the two groups (Kolmogorov-Smirnof test p = 0.952). Overall, the distribution and rate of the cell mortality was very similar in wells with or without beads during the whole period of the experiment. Therefore, the high death rate at the beginning of the cell culture is not due to the lack of activation. It could be rather due to the stress related to the transition from *in vivo* to *in vitro* culture.

**Figure 3:**
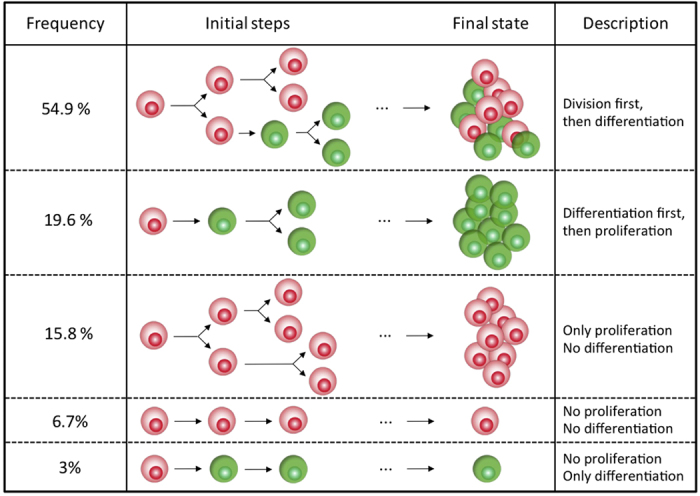
Different coexisting scenarios were observed during the first hundred hours suggesting that there is no pre-established sequence of events.

Surviving cells in micro-wells without activation beads did not divide and did not differentiate demonstrating that stimulation is essential for both processes. Therefore, we only considered individual cells that survived in the presence of at least a single bead. After a lag period of varying length, the majority of these cells started to proliferate and differentiate. Cell death also occurred sporadically at different stages of the experiment. In order to determine if differentiation occurs in a predetermined way, we recorded the cell number and phenotype in each individual micro-well every hour. Each clone displayed a different proliferation and differentiation dynamics (Fig. 3). More than 50% of the cell clones were composed of varying ratios of GFP+ and GFP– cells. About 20% of the clones were composed of only differentiated cells and 15% were pure GFP–. Interestingly, about 10% of the initial cells remained mitotically inactive in spite of the contact with the activation bead. Among them, some differentiated and others did not (Fig. 3).

In addition to the clones described above, we could observe micro-wells that randomly received a single starting cell and two or more activation beads. These cells had more chance to interact with the beads and received a stronger stimulus than those with a single bead. Nevertheless, no significant difference was observed between the dynamic proliferation/differentiation behaviors of the two categories of cells (Fig. 2).

In summary, all combinations of clonal differentiation/proliferation scenarios were simultaneously observed. Importantly, more than 50% of the clones were mixed with varying proportions of differentiated and undifferentiated cells, indicating that daughter cells within the same clones showed divergent proliferation and differentiation patterns. This observation suggests that the decision to differentiate is taken by individual cells according to a stochastic rather than deterministic mechanism and independently of the decision to divide or not.

### The unequal contribution of clones to the population increases with time as quantified by the Gini coefficient

**Figure 4:**
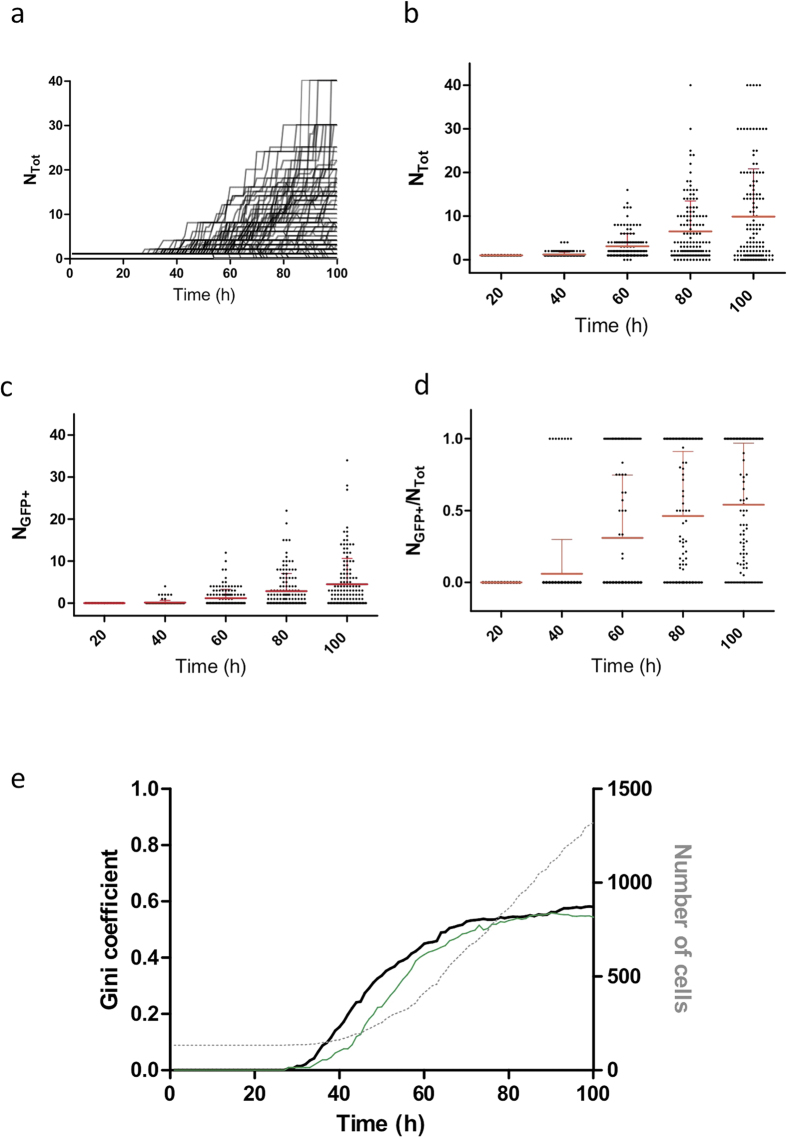
Evolution of the Gini coefficient and the population size in the *in vitro* experiment. (**a**) Individual proliferation curves show highly variable cell division rates. (**b**) Total number of cells per clone at five different time points during the experiment. (**c**) Number of GFP+ cells per clone at the same five different time points during the experiment. (**d**) Ratio of GFP+ to total number of cells per clone at the five time points of the experiment. Each point represents a single clone. The means and standard deviations are indicated with a thick and thin red bands respectively. (**e**) Evolution of the Gini coefficient calculated on the basis of the total cell number (black bold line) or GFP+ cells only (thin green line) in the cell population. Both curves increase rapidly between 30 h and 70 h and remain almost stable during the lasts stage with a final value of 0.581 and 0.544 at the end of the experiment. Interestingly, the total number of cells keeps increasing at the same rate (dashed line).

We observed substantial heterogeneity in the proliferation capacity of the clones. The time to the first division after stimulation varied from 15 to more than 100 hours. The subsequent cell cycles also varied in length, but much less than the lag period. In addition, cell death occurred sporadically during the whole period of the experiments leading to the extinction of more than 20 clones. By the end of the experiment, some micro-wells contained more than 30 cells while the majority less than 12. This means that only a small fraction of the founder cells contributed the majority of the cells to the final population defined as the total number of living cells at the end of the experiment. Such unequal contribution, that we chose to call Pareto-like effect, is frequently observed in nature or in social and economic life. A suitable parameter to measure this inequality is the Gini coefficient. The Gini coefficient varies between 0 and 1. It is equal to 0, if the population is composed by cells derived from each initial cell at equal proportion. On the other hand, the Gini coefficient is equal to 1 if all cells of the population descend from a single founder. In order to evaluate how the unequal contribution of the founder cells is established we calculated the Gini coefficient at every hour. As shown on the Fig. 4, the Gini coefficient started to increase concomitantly with the cell divisions. However, about 70 hours after the beginning of the experiment the increase of the Gini coefficient slowed down and appeared to converge to a value between 0.55 and 0.6 with a final value of 0.581 at the end of the experiment at 100 h. However, as indicated on the Fig. 4, during this final period the population continues to increase steadily. This tendency indicates that the unequal clonal contribution to the final population is essentially established during the first few cell divisions. We have separately calculated the Gini coefficient for the differentiated GFP+ cells only. We found that it increased following a similar dynamics and reached the value of 0.544 by the end of the experiment (Fig. 4). This value is almost identical than that found for the whole population and indicates that the undifferentiated GFP– and differentiated GFP+ cells follow a similar proliferation dynamics.

### A stochastic mathematical model can explain the contribution of proliferation and cell death to the Pareto-like effect

**Figure 5:**
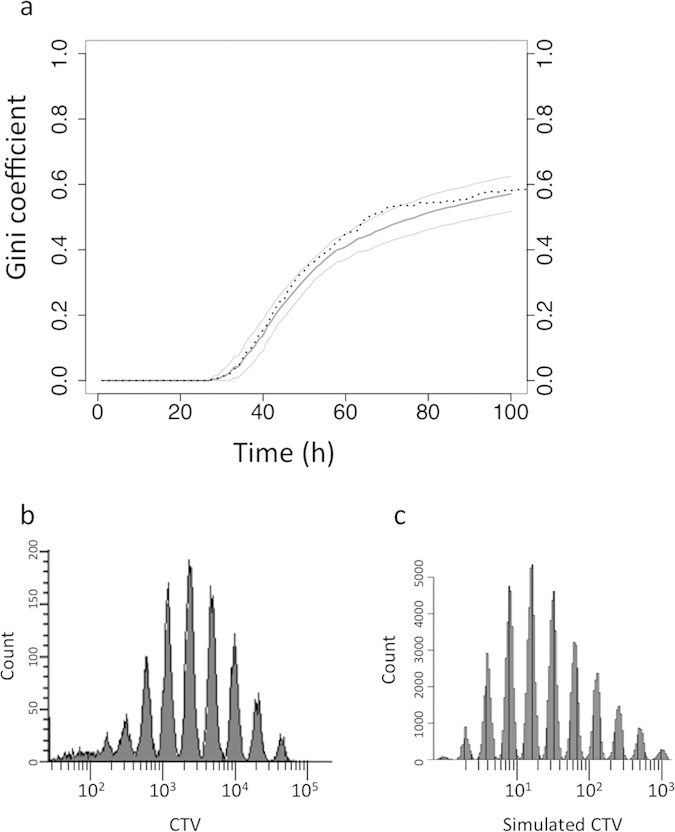
Evolution of the Gini coefficient and cell proliferation in the model. (**a**) The Gini coefficient in the model shows similar evolution (bold line) as in the experiment (dotted line). The +/– twice the standard deviation interval calculated on the basis of 100 simulations is shown by thin lines. (**b**) Cell proliferation profile obtained experimentally using CellTrace Violet (CTV)-fluorescent dye shows heterogeneous proliferation profile. The diagram represents the number of cells (Count) as a function of CTV fluorescence intensity (in arbitrary units). (**c**) The simulated proliferation profile is similar to that obtained experimentally.

Although it is clear that both differential proliferation and death rates may lead to a Pareto-like effect in the cell population, the simple description of the experimental system is insufficient to determine the relative contribution of these two potential sources. In order to address this issue, we designed a computer model, where each parameter can be independently manipulated. The model allows the simulation of the proliferation, death and differentiation of the cells based on a stochastic decision-making mechanism. Each cell can either, divide, die, differentiate or remain unchanged. The probabilities of these cell-fate events are the input parameters; they can be varied. In the basic version, we used probability values determined on the basis of the experimentally measured frequencies. Once the cell fate is chosen, the timing of the event is determined by drawing randomly from the experimentally determined distribution of the corresponding event. Since the first cell cycle was significantly longer than the subsequent cell cycles and the probability of the cell death and differentiation during this period was also significantly different, the probabilities were calculated separately for the first cycle (first event) and all subsequent cycles (subsequent events). We simulated simultaneously 133 cells in every run and we performed 100 independent runs. The Gini coefficient was calculated and plotted against the time in the same way as we did for the experimental values. The evolution of the median curve + /– with the 2 x standard deviation interval shown on the Fig. 5a is very close to the experimentally determined one. The median of the Gini Coefficient is equal to 0.6 at the end of the simulation and is almost equal to the experimentally measured value. In addition, our model was able to reproduce a similar profile of proliferation assay using dye labeling and flow cytometry on bulk culture (Fig. 5b,c). Therefore, the model based on a stochastic fate-determination mechanism can faithfully reproduce the experimentally observed Pareto-like effect when the input probabilities are calculated on the basis of the observed frequencies.

**Figure 6:**
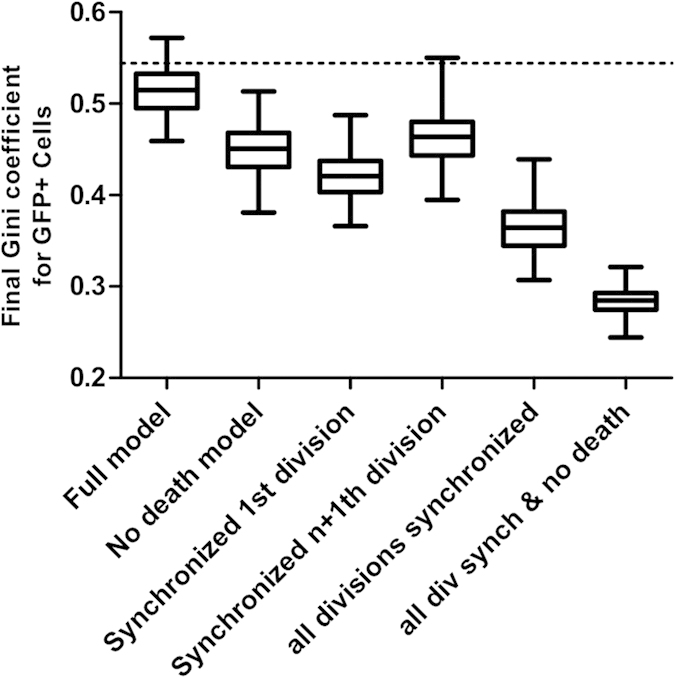
Variation of the Gini coefficient depending on the stochastic or deterministic nature of the input parameters. The average values of the Gini coefficients and their variations calculated under different conditions of simulation at 100 h are shown by boxplots. The horizontal dotted line shows the experimental value. The Kruskal-Wallis test (non-parametric ANOVA) on the 6 groups revealed that the medians varied significantly with a p-value < 0.0001. The Gini coefficient calculated in the full model is significantly higher than in the other models (p < 0.001) using the Dunn’s multiple comparison test.

In order to determine the relative contribution of the cell division and death probabilities to the Pareto-like effect, we performed simulations using fixed input parameters. First, we suppressed the cell death. Second, we used a constant first cell cycle length leading to synchronized first division of all cells. Then we tested the effect of constant cell-cycle length in the subsequent cell divisions followed by the synchronization of all cell divisions. Finally, we combined the first and the fourth cases, i.e. all divisions were synchronized and the cells were immortal (see Supplementary Table S1). We performed 100 runs for each case and calculated the average Gini coefficients with their standard deviations at the end of the simulation. Figure 6 show that each of the individual parameters contributes significantly to the Pareto-like effect (p < 0.001). When all three parameters were assigned a fixed value, the Gini coefficient dropped to a very low level. This observations show that the stochastic nature of the cell division and death together are the major causes of the unequal contribution of the individual clones to the final population. The remaining weak Pareto-like effect is presumably due to the variable time of cell differentiation that resulted in a modest but measurable difference in the doubling time of individual clones.

## Discussion

In this study, we have quantified the proliferation, differentiation and death rates of individual CD4+ T-cell clones after *in vitro* stimulation. We have observed that the high death rate during the first 24 hours following the isolation of the cells is independent of the TCR stimulation by the anti-CD3 and anti-CD28 antibody-coated beads. The initial high rate of cell death in *in vitro* experiments is traditionally attributed either to the overstimulation or the insufficient stimulation of the cells^12^. Our observations show that the cell death is independent from the TCR stimulation. We assume that the major cause of the high mortality may be associated to the cell stress induced by the manipulation and culture. The slow recovery from the stress may also explain why the lag before the first division of the surviving cells varied so widely. As expected, physical contact between the cell and an activating bead was mandatory for proliferation and differentiation. However, we could clearly observe in our time-lapse records that a permanent contact was not necessary. After a lag period of varying length, the cells started to proliferate and differentiate. We observed all possible combinations of proliferation and differentiation events (Fig. 3) that resulted in individual clones with only undifferentiated (GFP–), differentiated (GFP+ ) and mixed cells. The latter type represented more than the half of the clones. We also observed cells that acquired the GFP+ phenotype without cell division. These finding show that differentiation may occur independently of cell division, and more specifically, without asymmetric division. It is clear from these observations that there is no pre-determined order for division and differentiation; all options are possible and the timing of differentiation is not determined neither by the stimulation nor the proliferation rate.

Although there is no correlation between the cell divisions frequencies and the lag period before the first division, the clones grow at different rates. This is essentially due to the variation in the cell-cycle length of individual cells and sporadic cell death. As a result, 100 hours after the beginning of the experiment some clones are composed of a large number of cells (>>40) while others become extinct or are represented by a few cells only. The majority of the final population is composed of cells from a small number of clones. We used the Gini coefficient to describe the unequal clonal contribution to the whole population. The value of the Gini coefficient is equal to zero if all individual cells contribute equally to the clonal composition of the final population. The Gini coefficient is equal to 1 if the final composition is made up of a single clone. In our experiment, we calculated a Gini coefficient equal to 0.6, which means that a small number of clones outgrew the others and dominate the final population. Indeed, the evolution of the Gini coefficient over time describes well the dynamics of the population. The Gini coefficient starts to increase sharply after the lag period when the first cells start to divide and reaches a plateau around the final value of 0.6. The clonal structure of the final population is established during this period and in spite of the continuous growth of the population size it remains essentially unchanged at later stages. There are several factors that could contribute to the establishment of the Pareto-like clone size distribution. First, the time between the stimulation to the first division varies widely between cells. The rate of cell divisions is also variable. This is a well-known phenomenon^13^ and one would expect that positive and negative deviations would equilibrate each other conferring a stable average proliferation rate. Presumably, this happens at later stages when the number of cells in the clones is large enough to stabilize the average proliferation rate. However, at earlier stages the number of cells per clone is small and the clone size is very sensitive to fluctuations of division rates and fortuitous cell death. After the first wave during the first 24 hours, cell death occurs sporadically at all stages and in all clone types. Some clones even become extinct after the first few divisions. This is the third factor that can impact the clone composition of the population at the early stages of proliferation. Overall, these findings suggest that the cell-fate decisions to divide and differentiate are taken separately by individual cells using stochastic mechanisms. The individual random decisions lead in a few days to a biased clonal composition of the whole cell population characterized by the dominance of a few clones.

In order to estimate the relative contribution of proliferation/differentiation/death to the Pareto-like distribution we designed a simple mathematical model based on stochasticity. The cells were assigned a separate probability to accomplish the first and subsequent divisions or to die. The actual values of the probabilities were calculated on the basis of the frequencies observed during the experiment. Differentiation also occurred as a chance event with a probability calculated on the basis of the observed frequencies and distributions. As an output, we calculated the evolution of the Gini coefficient during the population growth. Comparing the results of the simulations and the observations, we can see that a stochastic model of cell division and death can account for the Pareto-like distribution of the clonal composition. The model allowed us to determine the relative contribution of the first division timing, division rate and cell death by setting them respectively to fixed values or zero. The simulations show that all three factors contribute to the Pareto-like effect and none of them is determinant.

In conclusion, our experimental observations and the results of the computer modeling indicate that no complicated deterministic mechanisms are needed to explain the proliferation and differentiation dynamics of naive CD4 + cells *in vitro*. A simple stochastic mechanism is fully sufficient. Further studies are required to determine if the Pareto-like effect found in the clonal composition plays a role during cell proliferation and differentiation *in vivo*. In *in vitro* studies however, this phenomenon can introduce a significant bias in our understanding of biological mechanisms. Particular characteristics of a limited number of clones selected by the culture conditions and transmitted by epigenetic mechanisms through cell divisions can hide and be confounded with cellular mechanisms induced by the experimental treatment.

## Methods

### Mice

All procedures on animals were performed in accordance with the European guidelines for the protection of vertebrate animals used for experimental purposes (Directive 2010/63/EU of 22 Septembre 2010). All experimental protocols were approved by the ethics committee of Genethon. Transgenic mice with fluorescent Treg cells (Tg(TcraH-Y,TcrbH-Y)1Pas, Ptprc,Foxp3, Rag2) were generated and housed in our facility by crossing Foxp3-GFP-KI mice (B6.Cg-Foxp3tm1Mal/J) with Marilyn mice (B6.129-Ptprca Rag2tm1Fwa Tg(TcraH-Y,TcrbH-Y)1Pas/Pas)^7^. Animals were used in experiments at between 6 and 9 weeks of age.

### Cells and activation

CD4 + T-cells were harvested from spleen of Foxp3-GFP^+/+^ transgenic mice and sorted using magnetic beads as described^7^. CD4 + T-cells were activated with activation beads (Gibco – Life Technologies, Dynabeads Mouse T-Activator CD3/CD28) coated with anti-CD3 and anti-CD28 antibodies.

### Micro-grid cell culture

A polydimethylsiloxane (PDMS) micro-grid array (Microsurfaces, Australia) of 4096 micro-wells (50 μm-sided) was placed in a MatTek dish (MatTek Corporation, Slovak Republic). It was covered by complete Roswell Park Memorial Institute (RPMI) 1640 Medium Glutamax plus (RPMI; Gibco-Invitrogen, France) supplemented with 10% fetal calf serum (FCS; PAA Laboratories, Germany), 100 μg/mL penicillin, and 100 μg/mL streptomycin (both from Eurobio, France), 50 μM 2-mercaptoethanol (Gibco-Invitrogen), and 30 U/mL IL-2 (Prometeus Laboratories) and 20 ng/mL TGF-β (Miltenyi Biotec SAS, France). The suspension of activating beads (see above) was added at a concentration that allowed the highest possible number of micro-wells with a single bead. Then, the suspension of 1.5 × 10^4^ cells was added, again at a concentration to get high number of wells with a single cell. As a result of this procedure, a large number of micro-wells with a single bead and a single cell were obtained together with micro-wells with cells without beads or two or more beads. We define a clonal population as being the cells in a micro-well that had one cell at the beginning of the experiment. The cells were maintained at 37 °C, 5% CO_2_.

### Proliferation assay

The CD4 + T cells were labeled with 1 μM of CellTrace Violet (CTV) (Life Technologies) and cells were analyzed by flow cytometry (LSR II – BD Biosciences, Le Pont de Claix, France) as described by Neildez *et al.*^7^.

### Time-lapse microscopy

Time-lapse acquisitions were performed with the Biostation IM time-lapse microscope (Nikon Instruments, Europe). 99 field positions were recorded covering 20 micro-wells each. Images were acquired every hour for 100 hours in phase contrast, GFP fluorescence channel (505–550 nm bandpass filter) and Rhodamine fluorescence channel (560–605 nm bandpass filter) at 20 X magnitude.

### Image analysis

Images were analyzed using ImageJ 1.47 g 64-bits (Rasband, W.S., ImageJ, U.S. National Institutes of Health, Bethesda, Maryland, USA, http://imagej.nih.gov/ij/, 1997–2014.). The cells were counted manually at each step of the time-lapse to determine the total number of cells, the number of differentiated cells (GFP channel) and the number of dead cells (identified on the basis of their auto-fluorescence in the Rhodamine channel). Micro-wells containing a single cell and at least one activation bead were considered in the analysis; micro-wells containing no beads and one single cell were used as controls.

### Experimental distribution and frequencies

Three types of events were considered: cell death, cell division and cell differentiation. For each micro-well a first event was determined and its time associated. By compiling all the micro-wells, 3 frequencies and 3 time distributions were calculated for the first event. The same approach was used to determined and calculated frequencies and time distributions of the second event according to the type of the cell, GFP– or GFP+ (See Supplementary Fig. S1).

### Gini coefficient calculations

The Gini coefficient enables the quantification of the Pareto-effect. It is calculated on the basis of the cell numbers in each clone (x_1,_ x_2,_…x_N_) as a sum of all combinations of absolute differences between the cell numbers in two clones, |x_i-_–x_j_|, normalized by N and μ.

μ is the arithmetic mean :


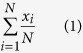


The Gini coefficient is given using the following formula [11]:


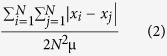


The Gini coefficient ranges from a minimum value of 0, when all clones contribute equally to the final cell population, to a theoretical maximum of 1 when the entire population is derived from a single founder cell and all other clones die out.

The Gini coefficient was calculated separately for GFP positive and GFP negative clones. We considered a clone as GFP positive if there was at least one GFP positive cell at the end of the experiment. For the sake of calculability, we considered that a GFP positive cell was initially in the micro-well. This consideration is only a mathematical artifice to avoid having a zero vector in the calculation of the Gini coefficient.

### *In sillico* model and simulations

Our model simulates individual cells that are able to divide, differentiate, die or remain unchanged. The cell fate is determined at each step using a decision tree. The model is divided into two parts: the first cell cycle of the founder cell and all subsequent cell cycles. In the first part, the model simulates the fate of the founder cell. We considered three possible fate decisions: division, differentiation or remaining unchanged till the end of the simulation. We calculated the probability of the first event on the basis of the observed experimental frequencies. Once the event is chosen for a given founder cell, the time to this particular event is assigned on the basis of its experimentally observed cumulative time distribution. We used the same process for every cell derived from the division of a founder cell except that cell death was added as a fourth possible event (See Supplementary Fig. S2). The probabilities and the time to the event were calculated on the basis of the cumulative distributions obtained experimentally on the cells after the first division of the founders.

In the model, each cell is considered as a vector containing parameters concerning its fate:


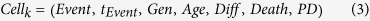


The parameters are:


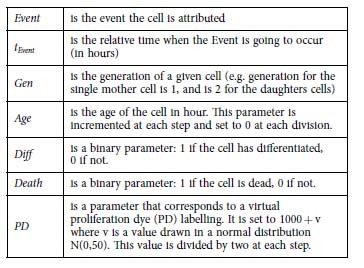


Each time point is associated to a matrix M that contains row vectors of the cells in the clone. Each step in the simulations represents an increment of 1 h. At each step, the matrix M is updated according to the previous state:


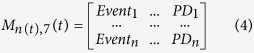


t is the time in hours.

The number of columns is 7 and the number of rows *n(t)* depends on the number of steps and on divisions.

The output is the number of total number living cells N_tot_ and the number of differentiated and living cells N_diff_ .

We simulated 133 clonal populations for 100 steps (100 hours) in each run and we performed 100 independent runs. We calculated the corresponding Gini coefficient either for all clones or only for clones containing differentiated cells.

The model was implemented using custom-made R (R Core Team (2013). R: A language and environment for statistical computing. R Foundation for Statistical Computing, Vienna, Austria. URL http://www.R-project.org/). Scripts are available on request (stockho@genethon.fr).

## Additional Information

**How to cite this article**: Cosette, J. *et al.* Single Cell Dynamics Causes Pareto-Like Effect in Stimulated T Cell Populations. *Sci. Rep.* **5**, 17756; doi: 10.1038/srep17756 (2015).

## Supplementary Material

Supplementary Information
