# Supplementary material for: Single Cell Dynamics Causes Pareto-Like Effect in Stimulated T Cell Populations
Source: Sci Rep. 2015 Dec 9;5:17756. doi: 10.1038/srep17756 (PMC4673432; doi:10.1038/srep17756)
Supplement: Supplementary Information [file srep17756-s1.doc]

**SINGLE Cell dynamics causeS PARETO-LIKE EFFECT IN STIMULATED T CELL POPULATIONS.**

Jérémie COSETTE^†1^, Alice MOUSSY^†3,2^, Fanny ONODI^1,2^, Adrien AUFFRET^1,2^, Thi My Anh NEILDEZ^3,2^, Andras PALDI^2,3^, Daniel STOCKHOLM^*3,2,1^

1. *Genethon, 1bis rue de l’Internationale, 91000 Evry, France*
2. *Unit INSERM INTEGRARE UMRS 951-UEVE-EPHE, 91000 Evry, France*
3. *Ecole Pratique des Hautes Etudes, Paris, France*

^†^ *These authors contributed equally to this work*

** Corresponding author:* e-mail: stockho@genethon.fr

**
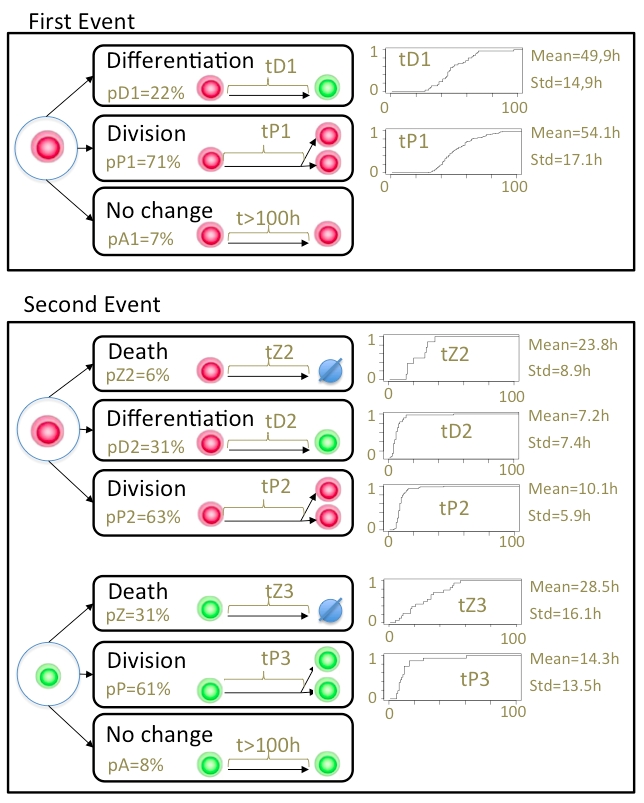
Supplementary Figure S1**. **Experimental frequencies and time distributions**. Three types of first events were considered: differentiation (D), cell division (P) and “no change” (A). If a cell died before division or differentiation, it was excluded from the calculation of the frequencies. In the second and subsequent events “death” (Z) was included. The probabilities (pD1, pP1, pA1 for the first events, pZ2, pD2, pP2 for the second event of non-differentiated and pZ3, pD3, pP3 for the second event of differentiated cells) were obtained from the frequencies calculated on the 133 clonal populations. A cumulative time distribution (tD1, tP1, tZ2, tD2, tP2, tZ3, tP3) is presented with the mean and standard deviation on the right.

**
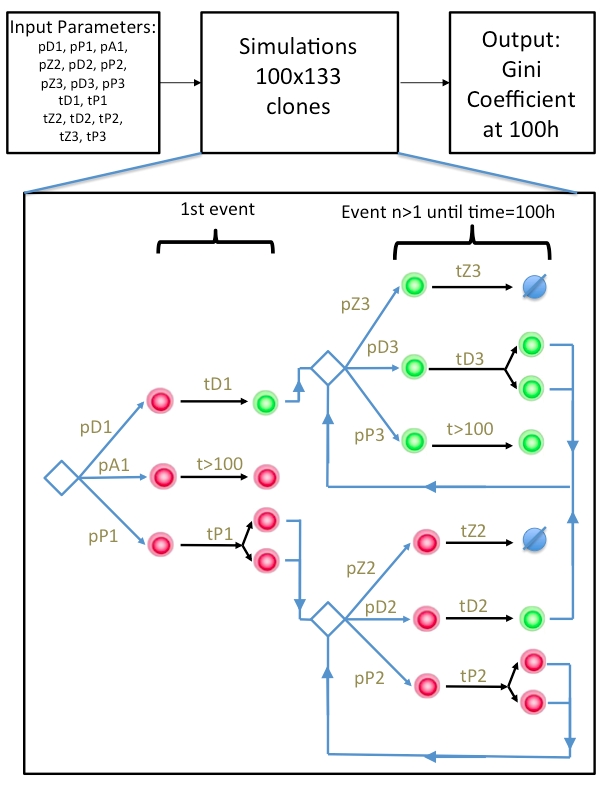
Supplementary Figure S2**. **The structure of the model** and the flow chart of the simulation of a clonal population is represented. Simulations rely on 9 probabilities (pD1, pP1, pA1, pZ2, pD2, pP2, pZ3, pD3, pP3) and 7 time distributions (tD1, tP1, tZ2, tD2, tP2, tZ3, tP3). Simulation starts with a non-differentiated cell. The first event (differentiation, division or “no change” until the end of the simulation at 100h) is chosen randomly according to the probabilities pD1, pP1 and pA1 (pD1+pP1+pA1=1). A new cell is created if a division occurs. The fate of each cell is then calculated according to the probabilities of the “second event” as shown on the right part of the diagram. A simulation step corresponds to 1h. In each run, 133 cell clones are simulated and the Gini coefficient is calculated for time=100h.

**Supplementary Table S1.** **Combinations of input parameters used for the simulations**. Initially, the simulations were performed using probabilities calculated on the basis of experimental observations (exp). In other the other variants of the model, one or several parameters were fixed to a constant value. (Const.). When the probability of cell death is fixed to “0”, the time parameter tZ becomes irrelevant, thus not applicable (NA).
